# Humanization of the mouse *Tert* gene reset telomeres to human length

**DOI:** 10.21203/rs.3.rs-3617723/v1

**Published:** 2024-01-05

**Authors:** De Cheng, Fan Zhang, Kenneth I. Porter, Shuwen Wang, Hui Zhang, Christopher J. Davis, Gavin P. Robertson, Jiyue Zhu

**Affiliations:** 1. Department of Pharmaceutical Sciences, College of Pharmacy and Pharmaceutical Sciences, Washington State University, Spokane, WA 99202, USA; 2. Department of Translational Medicine and Physiology, Elson S. Floyd College of Medicine, Washington State University, Spokane, WA 99202, USA; 3. Department of Pharmacology, Pathology, Dermatology, and Surgery, Pennsylvania State University College of Medicine, Hershey, PA 17033, USA

## Abstract

Telomeres undergo shortening with each cell division, serving as biomarkers of human aging, which is characterized by short telomeres and restricted telomerase expression in adult tissues. Contrarily, mice, featuring their longer telomeres and widespread telomerase activity, present limitations as models for understanding telomere-related human biology and diseases. To bridge this gap, we engineered a mouse strain with a humanized *mTert* gene, *hmTert*, wherein specific non-coding sequences were replaced with their human counterparts. The *hmTert* gene, encoding the wildtype mTert protein, was repressed in adult tissues beyond the gonads and thymus, closely resembling the regulatory pattern of the human *TERT* gene. Remarkably, the *hmTert* gene rescued telomere dysfunction in late generations of *mTert*-knockout mice. Through successive intercrosses of *Tert*^*h*/−^ mice, telomere length progressively declined, stabilizing below 10-kb. *Tert*^*h/h*^ mice achieved a human-like average telomere length of 10–12 kb, contrasting with the 50-kb length in wildtype C57BL/6J mice. Despite shortened telomeres, *Tert*^*h/h*^ mice maintained normal body weight and cell homeostasis in highly proliferative tissues. Notably, colonocyte proliferation decreased significantly in *Tert*^*h/h*^ mice during dextran sodium sulfate-induced ulcerative colitis-like pathology, suggesting limitations on cellular renewal due to short telomeres. Our findings underscore the genetic determination of telomere homeostasis in mice by the *Tert* gene. These mice, exhibiting humanized telomere homeostasis, serve as a valuable model for exploring fundamental questions related to human aging and cancer.

## Introduction:

Telomeres play key roles in the development of many human diseases, especially age-related disorders and cancer. Telomeres are replenished by telomerase, a ribonucleoprotein complex containing TERT, TERC, and accessory proteins ^1^. Humans are born with 10–15 kb telomeres and adults have 5–15 kb of telomeres ^2,3^. Telomerase activity is very low or undetectable in most adult human tissues. As a result, telomeres progressively shorten upon successive cell divisions and become exhausted in aged somatic tissues, triggering replicative senescence, and thereby functioning as an aging clock. Mutations in human telomerase genes lead to dyskeratosis congenita, a prototypical telomere biology disorder that presents as a multi-system syndrome with a broad spectrum of clinical manifestations, including aplastic anemia and cancer ^4^. Additionally, short telomere-induced replicative senescence in human cells also functions as a tumor-suppressing mechanism ^5^.

There exist important differences in telomerase regulation and telomere length among mammal species. In humans, telomerase expression is restricted to a small number of organs, such as testis, ovary, and thymus, and telomeres are exhausted in many aged tissues. However, a comparative study of over 60 mammals showed that many organisms, including mice, did not have telomere-mediated replicative aging ^6^. Telomerase expression in mice is less restricted, with most tissues expressing significant levels of Tert mRNA and telomerase activity ^7,8^. Laboratory inbred strains, such as C57BL/6, have long telomeres. Telomerase-null mice (*Tert* or *Terc*-KO) survive up to 6 generations with no discernible phenotypes in early generations, indicating that mice have adequate telomere reserves that are not exhausted for multiple generations without telomerase ^9^.

Mouse models of human diseases have become a central part of biomedical research. Laboratory mice provide the most experimentally accessible mammalian models that share genes, organs, and systemic physiology with humans. However, many mouse models fail to mimic human disease progression, posing translational challenges and limiting their use in human disease research. This may have contributed to the high failure rates of human clinical trials, particularly in oncology, predicating the need for improved preclinical data from animal models ^10^.

To develop a mouse model with human-like telomere homeostasis, we previously engineered a humanized *mTert* allele, *hmTert*, by replacing the 5′ intergenic region (5’IR), introns 2 and 6 of the *mTert* gene with their human counterparts in mouse embryonic stem cells (ESCs) ^11^. In the present study, we successfully obtained C57BL/6J mice with a germline *hmTert* gene. Tert mRNA expression and telomerase activity in these mice were distinct from those in wildtype C57BL/6J mice, but remarkably similar to those in humans. Importantly, the *hmTert* gene was able to rescue telomere deficiency when it was crossed into the 5^th^ generation of *mTert* knockout (KO) mice. Crucially, the introduction of *hmTert* allele led to a recalibration of telomere equilibrium length in these mice. Following successive intercrosses of *Tert*^*h*/−^ mice and subsequent incrosses of *Tert*^*h/h*^ mice, this new equilibrium settled at a range of 8–10 kb and 10–12 kb, respectively. The new equilibriums were notably shorter than that in wildtype C57BL/6J mice, but resembled human telomere lengths. This adjustment was also associated with a marked decrease in coloncyte proliferation following treatment with dextran sodium sulfate (DSS), suggesting that the shorten telomeres had a restrictive impact on the proliferative potential of somatic cells in these mice. In summary, the humanization of the *mTert* gene in C57BL/6J mice culminated in the development of a murine population that closely replicated human-like telomere homeostasis.

## Results:

### Telomerase regulation in mice with the hmTert allele

**Figure 1.**
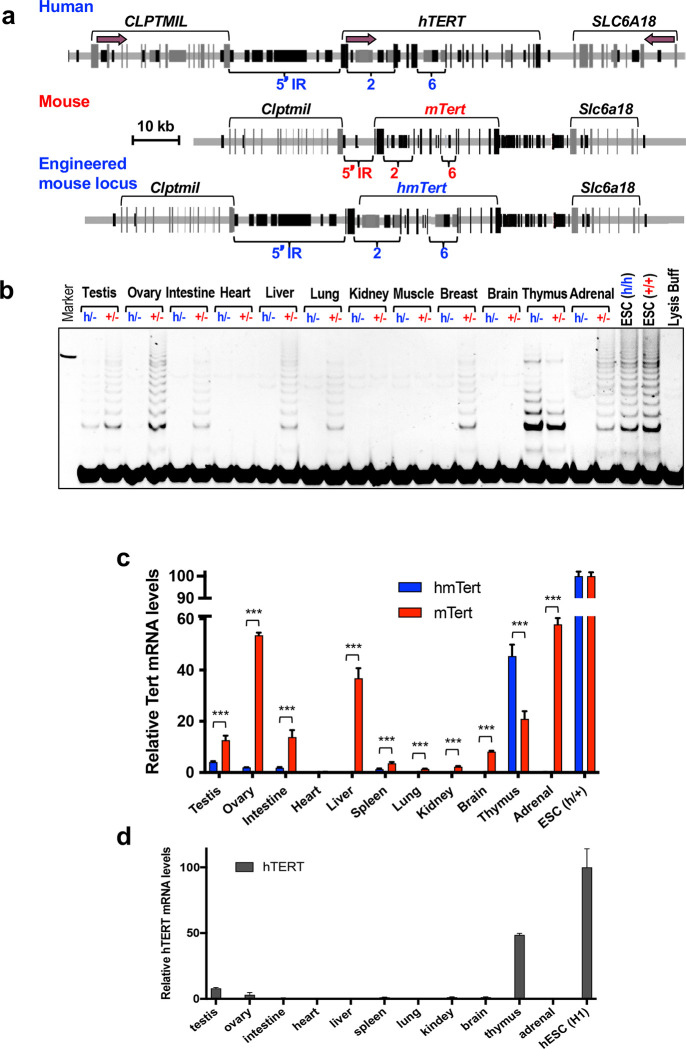
The *hmTert* gene and its expression in mice. **a**, Genomic maps of *hTERT*, *mTert*, and *hmTert* loci. Arrows indicate the directions of transcription. Vertical lines are exons; black and dark grey regions represent repetitive sequences, TEs, and VNTRs, respectively. Human and mouse 5’IR and introns 2 & 6 are labeled in blue and red, respectively. **b**, Telomerase expression in tissues from *Tert*^+/−^ and *Tert*^*h*/−^ littermates. Telomerase activities were determined by TRAP assay. 0.5μg protein extracts from 4-month-old mice were used except for thymus (0.12μg). +, h, and − refer to *mTert*, *hmTert*, and *mTert-KO* alleles, respectively. **c**, The expression of Tert mRNAs in adult mice. Tissues were collected from 4-month-old *Tert*^*h*/+^ mice. hmTert and mTert mRNAs were distinguished by using primers overlapping with the silent mutations in exon 2 of the *hmTert* allele. Mean and standard deviations (SDs) are shown. ***, p<0.001, two tailed student’s t test. **d,** hTERT mRNA expression in human tissues. Tert/TERT mRNA data were determined by qRT-PCR assay, normalized to 18S rRNA, and compared to those in *Tert*^*h*/+^ ESCs or human ESC H1 cells (1.0).

The *hmTert* gene, containing the human 5’IR (23-kb), introns 2 (11-kb) and 6 (5.5-kb) (Fig. 1a) was highly expressed in embryonic stem cells and stringently repressed upon differentiation ^11^. *Tert*^*h*/+^ mice were obtained and mated with *Tert*^+/−^ mice ^12^, generating mice of *Tert*^*h*/−^, *Tert*^+/−^, and *Tert*^*h*/+^ genotypes. As shown in Fig. 1b, telomerase activity was readily detected in the majority of adult tissues in a *Tert*^+/−^ mouse, yet it was present in a limited number of tissues in its *Tert*^*h*/−^ littermate. A direct comparison of mTert and hmTert mRNAs in a *Tert*^*hm*/+^ mouse showed that mTert mRNA was expressed in most organs, whereas high hmTert mRNA expression was found only in thymus (Fig. 1c). Relatively low levels of hmTert mRNA were detected in testis and ovary, and very low levels in intestine and spleen. The hmTert mRNA expression pattern was virtually identical to that of the hTERT mRNA in human tissues (Fig. 1d), indicating that the developmental regulation of the *hmTert* gene recapitulated that of *hTERT* in humans.

**Extended Data Figure 1.**
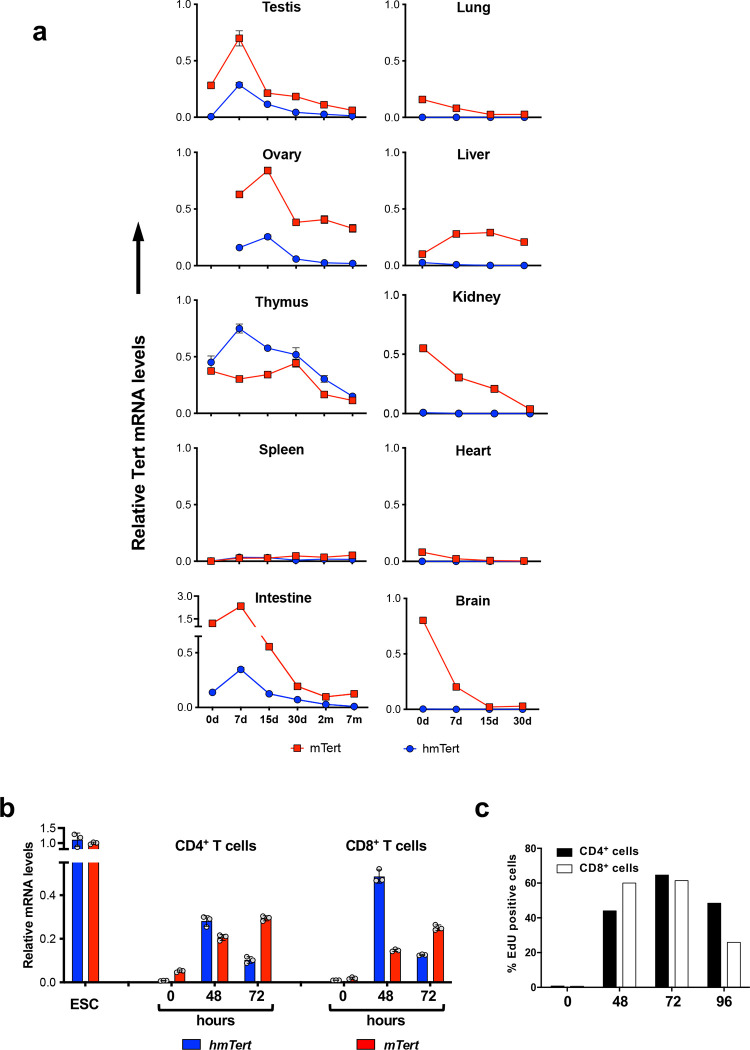
Developmental expression of the *hmTert* gene in mouse tissues. **a,** The expression of *hmTert* and *mTert* genes during post-natal development of *Tert*^*h*/+^ mice. **b,** hmTert and mTert expression during T cell activation. CD4^+^ and CD8^+^ T cells isolated from spleens of *Tert*^*h*/+^ mice were co-stimulated with CD3/CD28 antibodies and total RNAs were isolated. Tert mRNA data were determined by qRT-PCR assay, normalized to 18S rRNA, and compared to those in *Tert*^*h*/+^ ESCs (1.0). **c,** T cell proliferation. Resting T cells were stimulated with CD3/CD28 antibodies for 48–96 h and incubated with 10μM EdU for 1 h. Percentages of labeled cells were determined by flow cytometry.

During postnatal development, hmTert expression in testis, ovary, thymus, and intestine was more pronounced within the first two weeks of newborns and decreased with age in young mice (Extended Data Fig. 1a). Only thymus maintained significant hmTert expression. By comparison, mTert expression was high in most organs of newborns and decreased upon development, except for ovary, liver, and spleen, where high and low mTert mRNA levels were maintained in adults, respectively.

It was reported that hTERT expression progressively declined during T cell differentiation ^13^ and T cells rapidly upregulated hTERT mRNA to support robust cell division and differentiation ^14^. Consistently, resting mouse T cells expressed little hmTert mRNA and its level increase dramatically in CD4^+^ and CD8^+^ T cells following stimulation by CD3/CD28 antibodies for 48 and 72 hours (Extended Data Fig. 1b). mTert RNA was readily detected in resting T cells and its level also increased during T cell activation. The increase of hmTert and mTert RNAs correlated with EdU incorporation, and thus cell proliferation, in T cells (Extended Data Fig. 1c).

### Telomere lengths in Tert^+/−^, Tert^h/−^, and Tert-KO mice.

**Figure 2.**
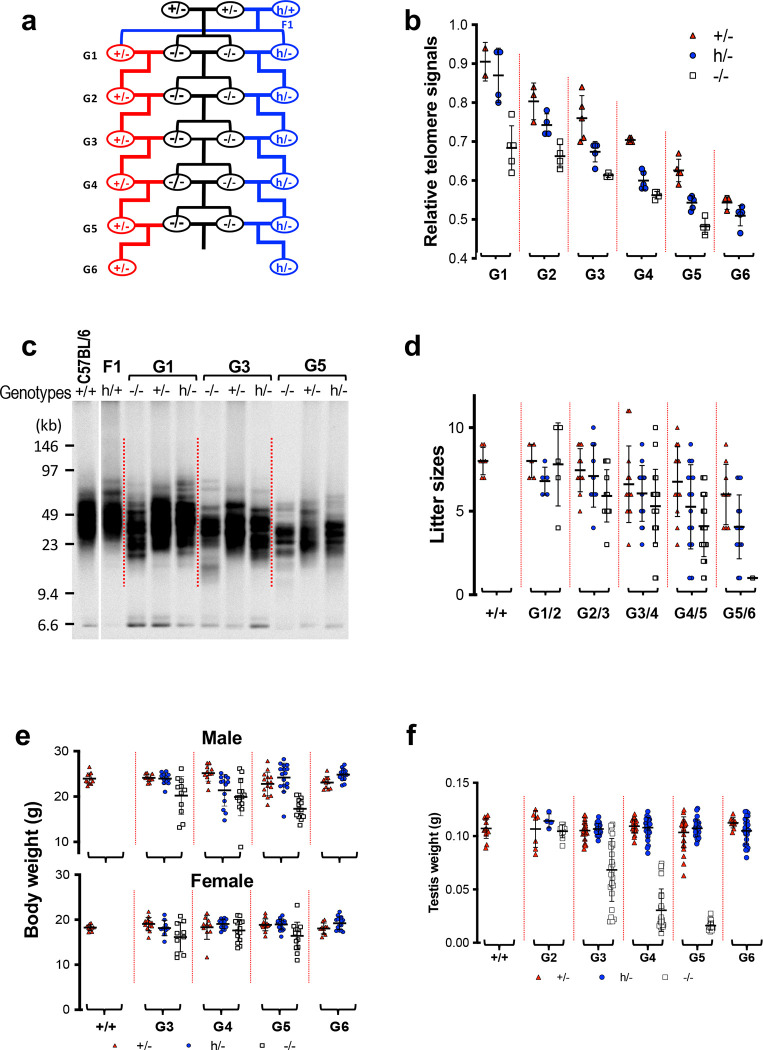

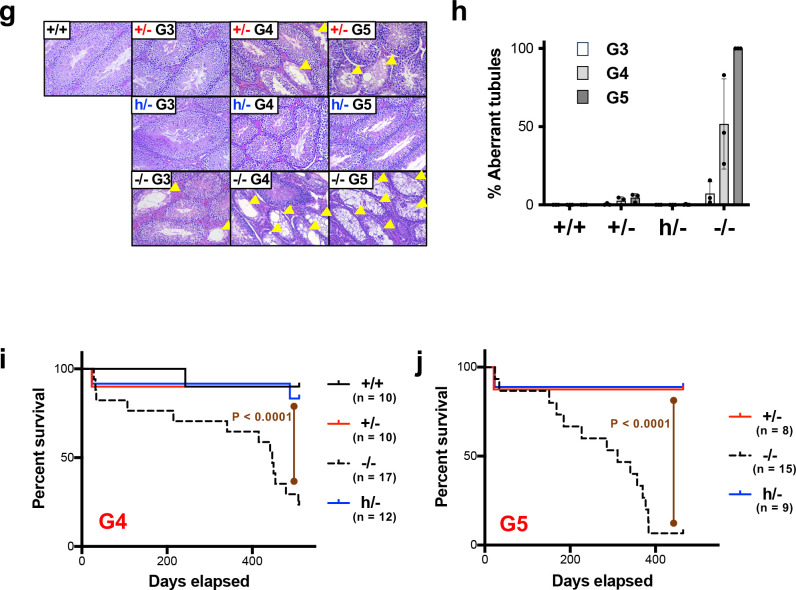
Functions of the *hmTert* gene in mice. **a,** Breeding strategy. Telomere length of splenocytes from 2-month-old *Tert*^+/−^, *Tert*^*h*/−^, and *Tert*^−/−^ mice were determined by Flow-FISH (**b**) and telomere restriction fragment (TRF) analysis (**c**). **b,** Telomere Flow-FISH. Telomere signals were detected by hybridization to FAM-(CCCTAA)_3_ oligonucleotide. Fluorescence signals were compared to that of wildtype C57BL/6J mice (1.0). **c,** TRF analysis. Splenocyte genomic DNAs were digested with *Hinf*I and *Rsa*I, followed by pulsed-field gel electrophoresis and Southern blotting. Positions of size markers are shown on the left (kb). **d,** Litter sizes of breeding between *Tert*^+/−^ and *Tert*^−/−^ (red), *Tert*^*h*/−^ and *Tert*^−/−^ (blue), *Tert*^−/−^ and *Tert*^−/−^ (black) mice. **e,** Body weight of male (upper) and female (lower) mice at 8-week of age. **f,** Testis weight of mice at 10–15-week age. **g,** H&E staining of seminiferous tubules in testes from *Tert*^+/−^, *Tert*^*h*/−^, and *Tert*^−/−^ mice. Yellow arrowheads indicate aberrant tubules. **h,** Average percentages of aberrant seminiferous tubules in testes from 3–5 mice in each group. **i** and **j,** Survival curves of mice with *mTert*, *hmTert*, and *mTert-KO* alleles. Mice were bred as shown in panel A. Kaplan-Meier survival curves of G4 (**i**) and G5 (**j**) mice are shown. P-values of survival curve comparisons were calculated using logrank test. Means and SDs are shown.

C57BL/6J mice have an average telomere length of approximately 50 kb. To reduce telomere length in mice with *hmTert* genes, *Tert*^*+/h*^ F1 mice were crossbred to *Tert*^+/−^ mice to produce *Tert*^*h*/−^ and *Tert*^+/−^ offspring, which were then bred with successive generations of *Tert*^−/−^ mice (Fig. 2a). Telomere length of these mice were examined by flow cytometry following fluorescence in situ hybridization (Flow-FISH) and telomere restriction fragment (TRF) analysis by Southern blotting (Figs. 2b–c). As the breeding of *Tert*^−/−^ mice resulted in shortened telomeres, similar decreases in telomere length were observed in both *Tert*^+/−^ and *Tert*^*h*/−^ mice over successive generations. In each generation, *Tert*^*h*/−^ mice had shorter telomeres on average compared to their *Tert*^+/−^ counterparts. In G6 *Tert*^*h*/−^ mice, the average telomere length dropped to roughly 50% of the length in wildtype mice, or ~25 kb. The litter sizes of *Tert*^+/−^×*Tert*^−/−^ and *Tert*^*h*/−^×*Tert*^−/−^ mattings declined over generations, while the litter sizes of homozygous *Tert*^−/−^ incrosses decreased even more dramatically (Fig. 2d). In several attempts, G6 *Tert*^−/−^ mice produced only one offspring which died prematurely, ultimately ending this breeding strategy. However, the reduction in telomere length in *Tert*^+/−^ and *Tert*^*h*/−^ mice did not negatively impact their overall health and well-being, as evidence by their normal body weight in G6 mice (Fig. 2e).

### Maintaining testis homeostasis by the hmTert gene.

Due to the impact of shorter telomeres on mouse fertility, we analyzed the testes of these mice. As illustrated in Figs. 2f–h, *Tert*^−/−^ mice showed testicular atrophy as well as a progressive loss of germ cells in seminiferous tubules starting from G3 mice and worsening in G4 and G5 mice, as previously reported in telomerase deficient mice ^15^. *Tert*^+/−^ mice also exhibited a low level of testicular defects in G4 and G5, but such defects were absent in *Tert*^*h*/−^ mice. These data indicate that both *mTert* and *hmTert* genes help preserve germ cells in the testis, with *hmTert* showing a slight advantage in preventing germ cell loss.

### Sustaining mouse lifespan by the hmTert gene.

Previous studies have shown that telomere deficiency can impact mouse survival and lifespan ^16,17^. Our results align with those findings, as shown in Figs. 2i–j, G4 and G5 *Tert*^−/−^ mice had a significantly shortened lifespan, with median survival of approximately 440 and 320 days, respectively. However, the majority of wildtype *Tert*^+/+^, G4 and G5 *Tert*^+/−^ and *Tert*^*h*/−^ mice lived past 500 days, indicating that the presence of a *mTert* or *hmTert* gene could sustain longevity even when telomeres were relatively short.

### Rescuing telomere dysfunction in Tert^−/−^ mice by the hmTert gene.

**Extended Data Figure 2.**
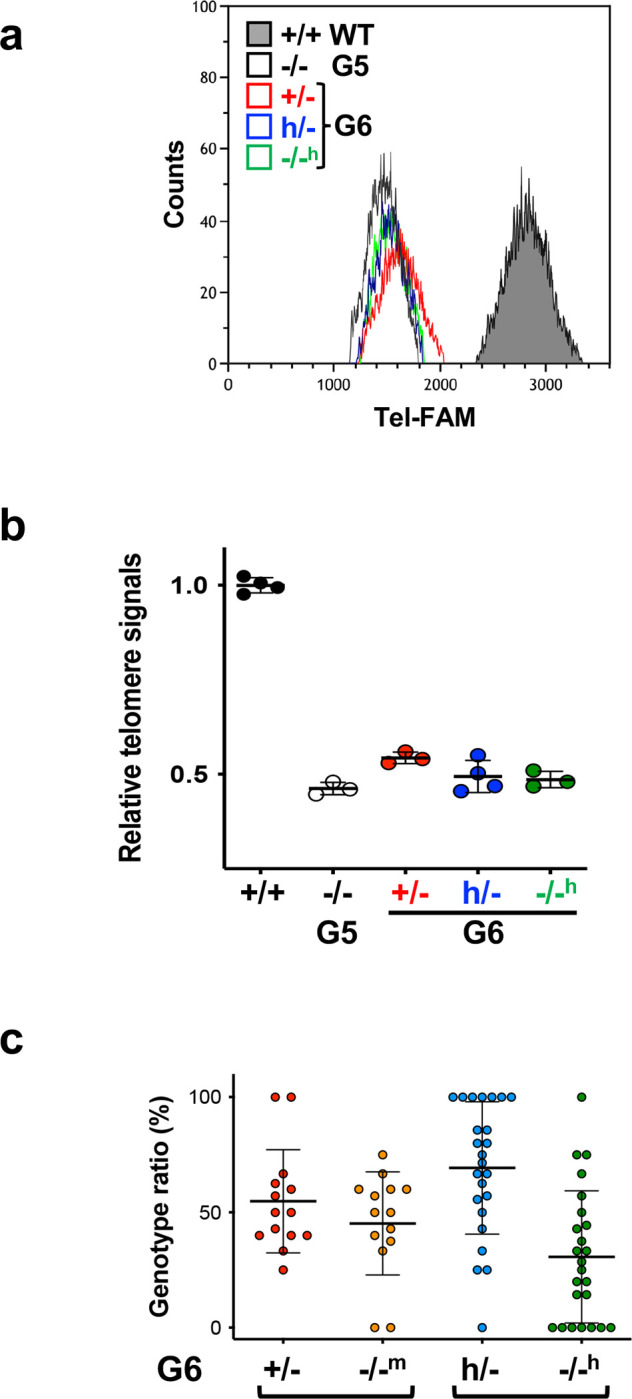
Telomere length and genotype ratios of G5 and G6 mice in Figure 2. **a** and **b,** Telomere length as determined by Flow-FISH. Telomere fluorescent signals of representative mice are shown in (**a**) and the data are summarized in (**b**). **c,** Genotype ratios of born offspring from G5 parents. Each data point represents one litter.

**Figure 3.**
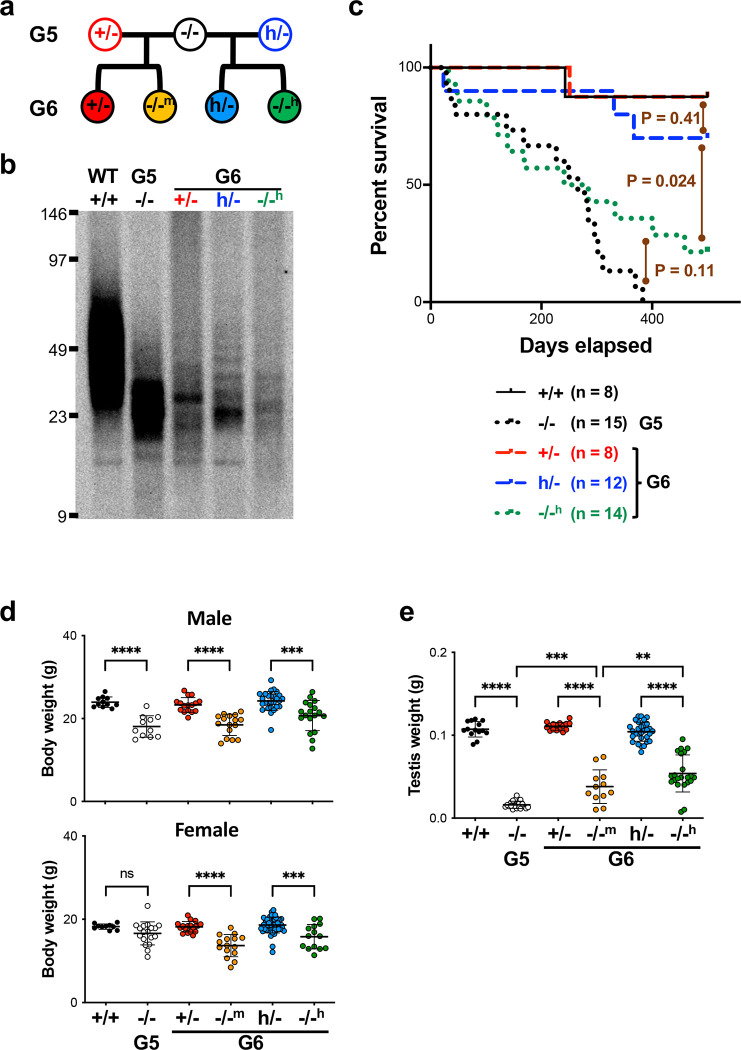

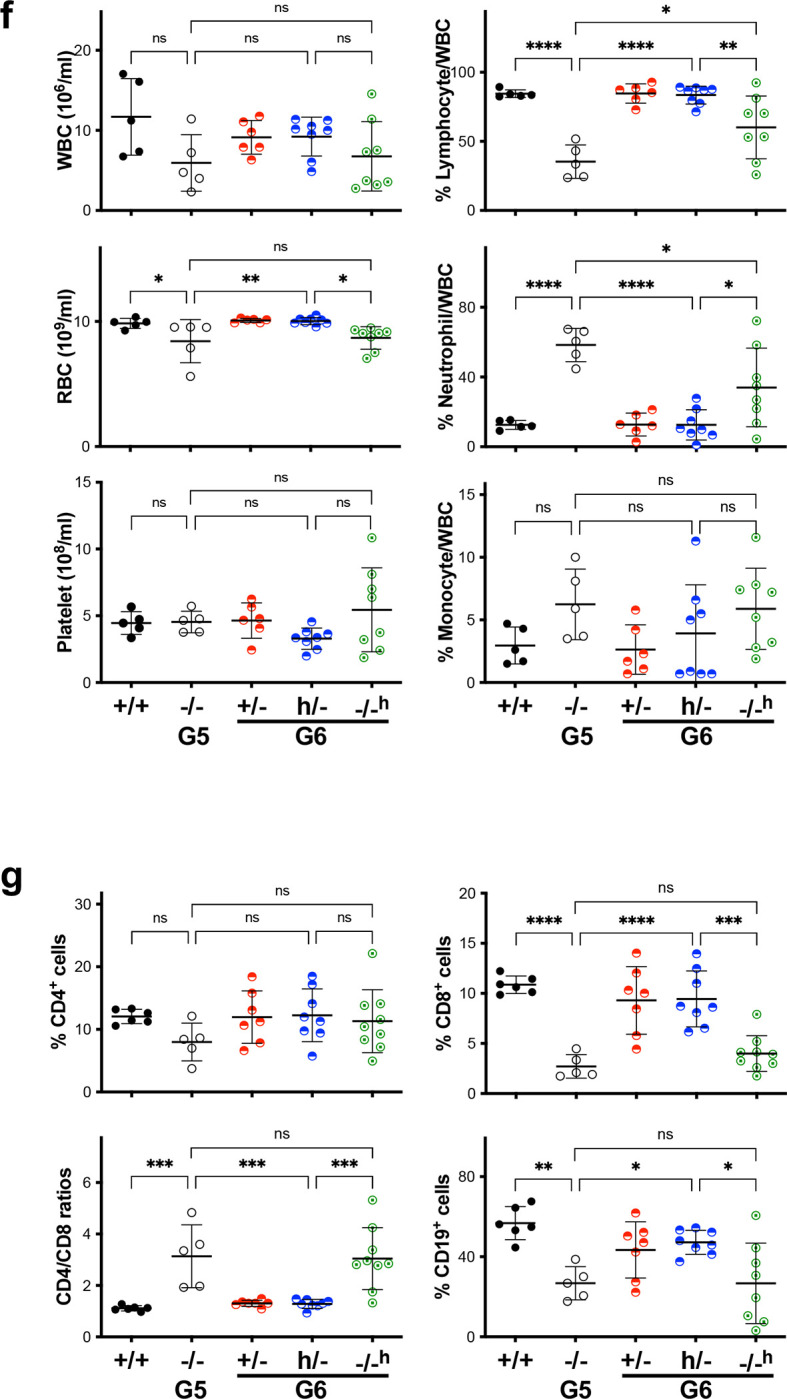

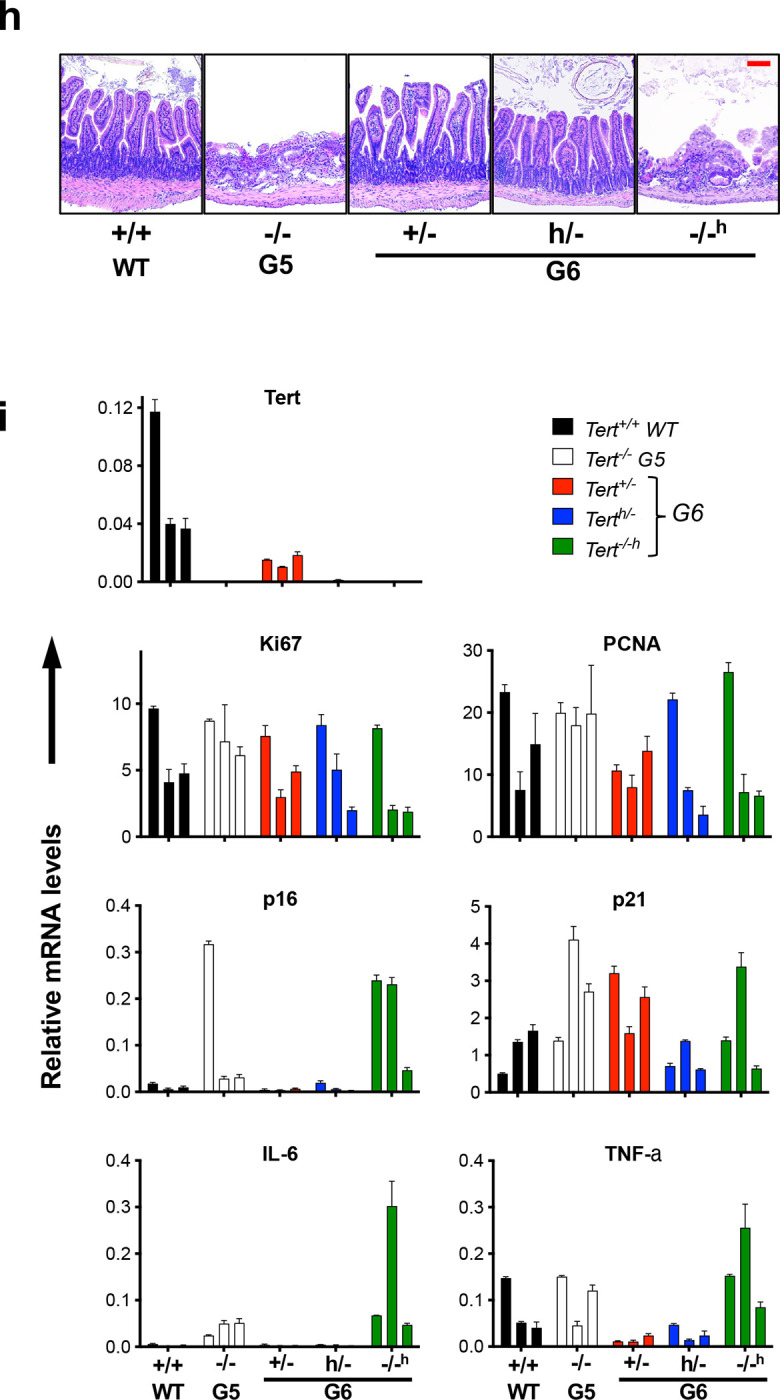
Comparisons of G6 mice with *mTert* and *hmTert* alleles. **a,** Mouse breeding scheme. **b,** TRF analysis of representative animals. Splenocyte genomic DNAs were digested with *Hinf*I and *Rsa*I, followed by pulsed-field gel electrophoresis and Southern blotting. **c,** Kaplan-Meier survival curves of mice. P-values comparing indicated paired curves were determined using logrank tests. **d,** Body weight of male (upper) and female (lower) mice at 8-week of age. **e,** Testis weight of mice at 10–15-week age. **f,** Whole blood cell counts in adult mice of 3–6 months by hematology analyses. **g,** Lymphocyte counts in peripheral blood. Cells were stained using antibodies and analyzed by flow cytometry. Means and SDs are shown. ns, not significant; **, P < 0.01; ***, P < 0.001; ****, P < 0.0001; one-way Anova. **h,** Histopathology of small intestines of adult mice of 8–10 months. The bar indicates 100 μm. **i,** The expression of genes regulating cellular senescence and proliferation in small intestine. Each column represents an individual mouse. Relative mRNA levels were determined by qRT-PCR and normalized to 18S rRNA. Means and SDs are shown.

Offspring inherit not only parents’ genotypes but also the lengths of their telomeres. G5 *Tert*^−/−^ mice displayed severe telomere dysfunction, evidenced by tissue dystrophy, reduced body weight, and a shortened lifespan. The next generation, denoted as G6 *Tert*^*−/−m*^ and G6 *Tert*^*/−h*^, was generated by crossing G5 *Tert*^−/−^ mice with G5 *Tert*^+/−^ and *Tert*^*h*/−^ mice, respectively (Fig. 3a). Examination of telomere length through TRF and Flow-FISH analyses indicated that the average telomere lengths in G6 *Tert*^*h*/−^ and *Tert*^+/−^ mice were comparable to, or slightly longer than, those of their G5 *Tert*^−/−^ parents and G6 *Tert*^−/−^ siblings (Fig. 3b and Extended Data Figs. 2a–b). However, despite these similarities, G6 *Tert*^*h*/−^ and *Tert*^+/−^ mice exhibited significantly extended lifespans compared to their G5 *Tert*^−/−^ mice (Fig. 3c). Notably, while all G5 *Tert*^−/−^ mice died within 383 days, three out of 14 G6 *Tert*^*−/−h*^ mice survived beyond the entire 460-day experimental period. This resilience could be attributed to the inheritance of short yet functional telomeres from their G5 *Tert*^*h*/−^ parents. Taken together, these findings suggest that the *hmTert* allele in *Tert*^*h*/−^ mice has the capacity to restore the shortest and likely most impaired telomeres, even in the context of their overall short average telomere length.

Further examination of genotypes among G6 progeny indicated that fewer G6 *Tert*^−/−^ offspring were born compared to their *Tert*^+/−^ and *Tert*^*h*/−^ siblings (Extended Data Fig. 2c), indicating that some *Tert*^−/−^ offspring died during prenatal development. Although G6 *Tert*^*−/−m*^ and G6 *Tert*^*−/−h*^ mice had reduced body and testis weights compared to their *Tert*^+/−^ and *Tert*^*h*/−^ littermates, respectively, their testes weighed significantly more than their G5 *Tert*^−/−^ parents (Figs. 3d–e). In addition, G6 *Tert*^*−/−h*^ mice had a slightly increased testis weight compared to G6 *Tert*^*−/−m*^ mice, suggesting that the *hmTert* gene was functionally similar to, or somewhat better than, the *mTert* gene for rescuing testicular defects. Overall, G6 offspring showed better general health compared to their G5 *Tert*^−/−^ parents, demonstrating the telomere function-restoring capacities of both the *hmTert* and *mTert* genes within a single generation.

### hmTERT Function in immune system.

**Extended Data Figure 3.**
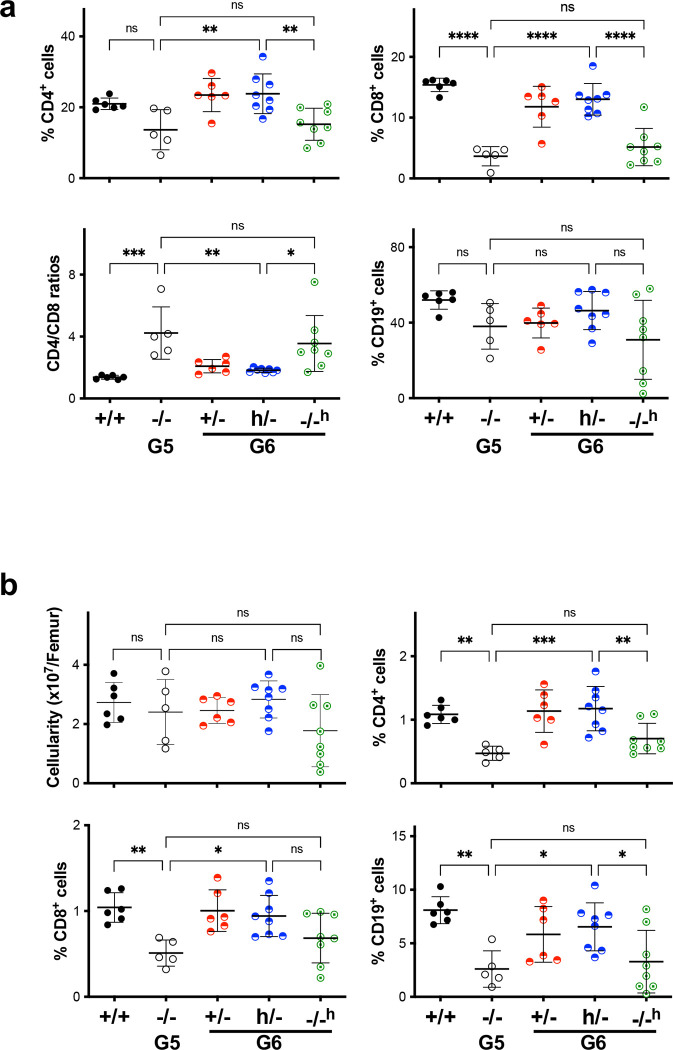
Hematopoietic cells in adult mice of 3–6 months. Mice were bred in Fig. 3a. **a** and **b,** Lymphocyte counts in spleen (**a**) and bone marrow (**b**). Cells were stained using antibodies and analyzed by flow cytometry. Each data point represent one animal. Means and SDs are shown. ns, not significant; *, P < 0.05; **, P < 0.01; ***, P < 0.001; ****, P < 0.0001; one-way Anova.

Previous studies have shown that hematopoietic cells’ proliferative capacity was compromised in telomerase-deficient mice, and human short telomere syndromes cause anemia, decreased erythropoiesis, and T cell immunodeficiency ^18–20^. Consistent with earlier reports, peripheral blood from G5 *Tert*^−/−^ mice exhibited a slight decrease in white blood cell (WBC) counts, a statistically significant reduction in red blood cell (RBC) counts, and normal platelet numbers (Fig. 3f). Among WBCs, there was a marked decrease of lymphocytes observed in G5 *Tert*^−/−^ mice, accompanied by corresponding increases in the ratios of neutrophils and monocytes. Both G6 *Tert*^+/−^ and *Tert*^*h*/−^ mice had blood cell counts similar to wildtype mice. The lymphocyte and neutrophil cell percentages within WBCs of G6 *Tert*^*−/−h*^ mice were between those of wildtype mice and their G5 *Tert*^−/−^ parents. G5 *Tert*^−/−^ mice had a somewhat reduced percentage of CD4^+^ T cells and dramatically reduced CD8^+^ T cells, leading to a significant increase of the CD4/CD8 T cell ratio (Fig. 3g). Additionally, CD19^+^ B cells decreased in G5 *Tert*^−/−^ mice. All these cell counts in G6 *Tert*^+/−^ and *Tert*^*h*/−^ mice were restored to the levels found in wildtype mice. Further analyses of T and B cell counts in spleen and bone marrow revealed similar changes in G5 *Tert*^−/−^ mice and *Tert*^+/−^ and *Tert*^*h*/−^ offspring (Extended Data Fig. 3). In short, our data indicate that the *hmTert* gene effectively rescued blood cell defects in G5 *Tert*^−/−^ mice within one generation.

### hmTERT Function in small intestine.

The gastrointestinal tract is another high proliferation tissue that is affected by telomere dysfunction ^21^. Depletion of the intestinal epithelial crypts and severe villus atrophy were observed in small intestines of older G5 *Tert*^−/−^ (≥ 8 months), but not in G6 *Tert*^+/−^ and *Tert*^*h*/−^ mice (Fig. 3h). The intestinal lesions probably contributed to the loss of body weight and overall poor health of *Tert*^−/−^ animals due to decreased nutritional absorption.

The intestinal defects are likely a consequence of cell cycle inhibition and cellular senescence induced by telomere dysfunction. Therefore, the expression of genes involved in cell proliferation was examined. Tert mRNA was readily detected in the intestines of *Tert*^+/+^ and *Tert*^+/−^ mice, but not in those of *Tert*^*h*/−^ or *Tert*^−/−^ mice, confirming that the *hmTert* gene is strictly regulated in adult tissues (Fig. 3i). Markers of cell proliferation, Ki-67 and PCNA, and the cell cycle inhibitor p21 were found in the intestine of all genotypes. Senescence-associated genes, *p16*^*Ink4a*^ and *IL-6*, were upregulated in G5 *Tert*^−/−^ and G6 *Tert*^*−/−h*^ mice, but not in any mice with *mTert* or *hmTert* genes. TNF-α, another pro-inflammatory cytokine secreted by senescent cells, appeared to be expressed in mouse intestines of all genotypes. Therefore, our data suggest that cellular senescence occurred in *Tert*^−/−^ intestines with significant telomere dysfunction but was suppressed by the presence of the *hmTert* gene in this tissue.

### Telomere shortening during intercrosses of Tert^h/−^ mice.

**Figure 4.**
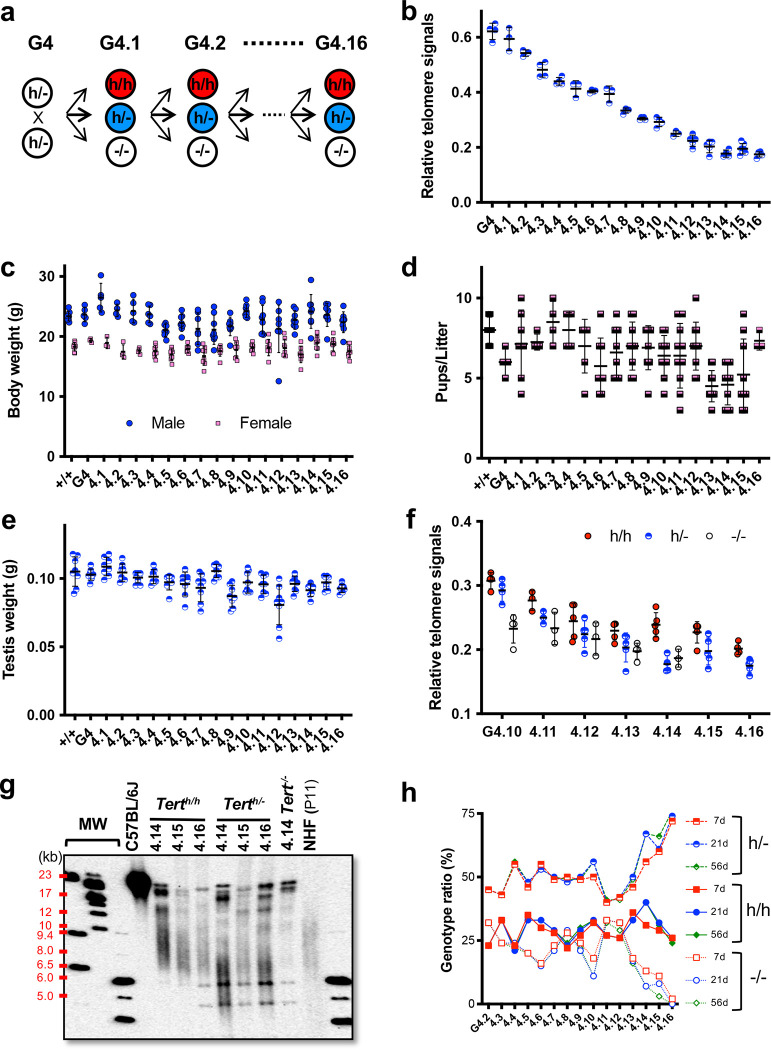
Telomere length homeostasis in mice during *Tert*^*h*/−^ intercrosses. **a,** Breeding strategy. *Tert*^*h*/−^ progeny from G4 *Tert*^*h*/−^ parents were intercrossed. **b,** Telomere length as determined by Flow-FISH. Splenocytes from 2-month-old mice were used for the analyses. **c,** Body weight of 8-week-old male and female mice. **d,** Litter sizes. **e,** Testis weight of mice at 10–15-week age. **f,** Flow-FISH comparing telomere lengths of *Tert*^*h/h*^, *Tert*^*h*/−^, and *Tert*^−/−^ littermates. **g,** TRF analysis. Splenocyte genomic DNAs were digested with *Hinf*I and *Rsa*I, followed by 0.6% Agraose gel electrophoresis and Southern blotting. Sizes are indicated on the left (kb). MW, molecular weight marker. NHF (P11), passage 11 normal human foreskin fibroblasts. **h,** Genotype ratios of progeny in intercrosses at 7, 21, and 56 postnatal days. Means and SDs are shown in panels B-F.

Despite its restricted expression in adult tissues, the *hmTert* gene rescued telomere dysfunction in G5 *Tert*^−/−^ mice with relatively short average telomeres of ~25-kb. Our next objective was to determine the telomere length setpoint influenced by the *hmTert* gene. To this end, G4 *Tert*^*h*/−^ mice were continuously intercrossed for 16 generations, from G4.1 to G4.16 (Fig. 4a). Using Flow-FISH, we monitored telomere length of *Tert*^*h*/−^ mice in splenocytes at each generation. As depicted in Fig. 4b, the average telomere length of *Tert*^*h*/−^ mice decreased from 60% to 18% of that observed in wildtype mice from G4 to G4.14, eventually stabilizing at 18–19% in the last three generations (G4.14 to G4.16). Throughout the breeding generations, both male and female *Tert*^*h*/−^ mice maintained body weights similar to those of wildtype mice (Fig. 4c). During this process, litter sizes varied, but were largely maintained (Fig. 4d). Male mice also maintained their testis weight (Fig. 4e). Fig. 4f compares the average telomere length of all three genotypes in each generation, from G4.10 to G4.16. It demonstrates that *Tert*^*h/h*^ mice in general had longer telomere than their *Tert*^*h*/−^ siblings, and that *Tert*^−/−^ mice consistently exhibited the shortest telomeres across generations. Overall, our data indicated that average mouse telomeres could be shorten to below 10 kb without affecting their overall health, at least at a young age, as long as they have the *hmTert* gene.

Telomere length in later generations of mice was also verified using TRF analysis (Fig. 4g). Two types of telomeres were found in these mice: discrete bands of variable sizes and intensities between 15 and 20kb, and shorter human-like telomere smears. The average lengths of the telomere smear were 8–9 kb in the G4.16 *Tert*^*h/h*^ mice and about 7 kb in the *Tert*^*h*/−^ mice. In a G4.14 *Tert*^−/−^ mouse, the telomere smear was much less apparent. Fig. 4h shows that, from G4.2 to G4.12, approximately 50% of total born mice were of heterozygous *Tert*^*h*/−^ genotype, while homozygous *Tert*^*h/h*^ and *Tert*^−/−^ mice each accounted for about 25% of total progeny, following Mendelian genetics. However, there was a sharp decline in the numbers of *Tert*^−/−^ mice born from G4.13 to G4.16. The few *Tert*^−/−^ mice that were born were small and die at young ages. These data indicated that short telomeres in late-generation *Tert*^−/−^ embryos could no longer sustain mouse development.

### Maintaining stable human-like telomeres in homozygous Tert^h/h^ mice.

**Figure 5.**
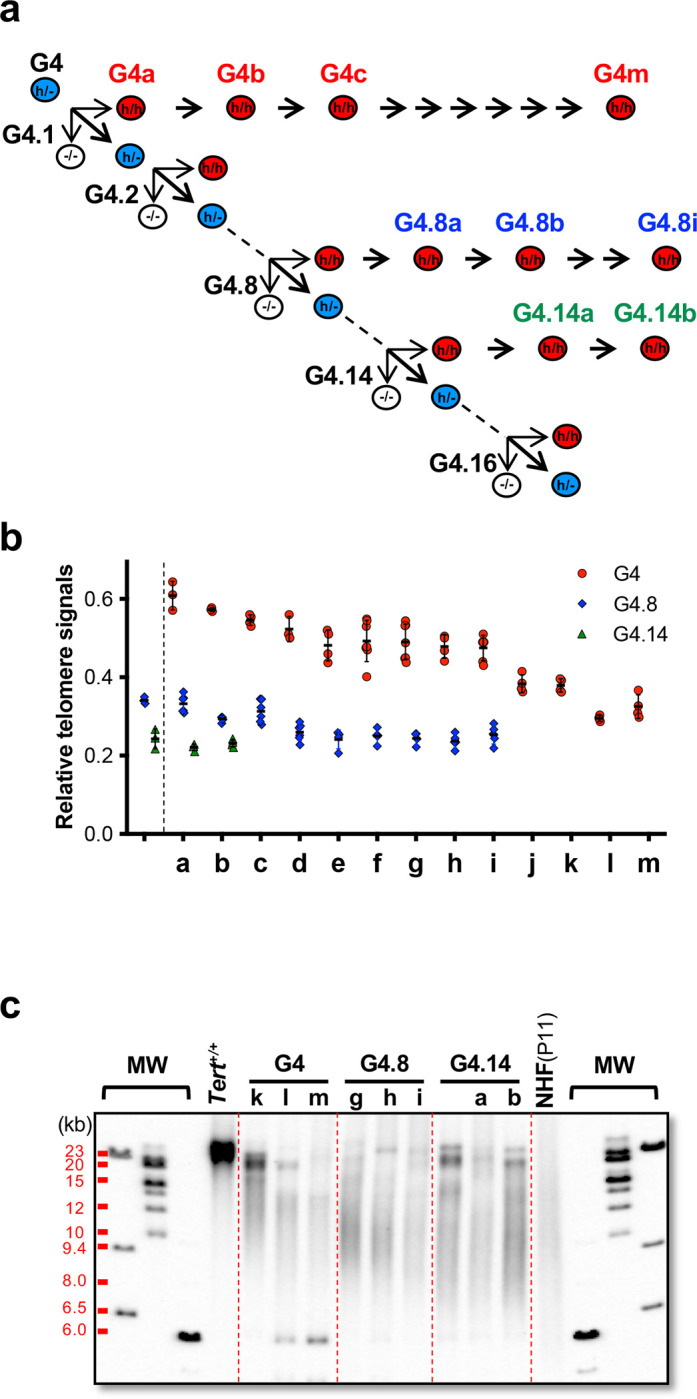

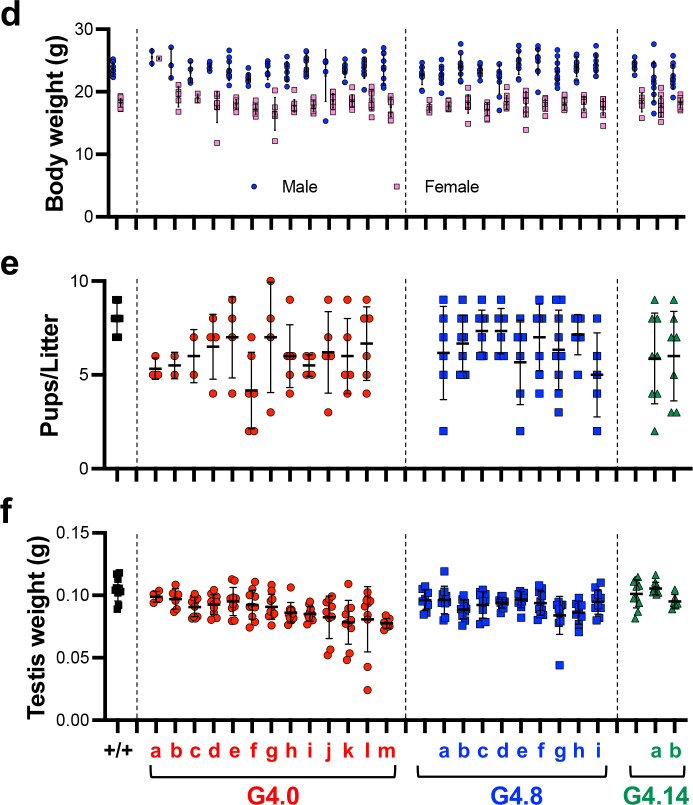
Telomere length homeostasis during incrosses of *Tert*^*h/h*^ mice. **a,** Breeding schemes. *Tert*^*h/h*^ progeny from G4, G4.8, and G4.14 *Tert*^*h*/−^ parents were successively incrossed. **b,** Relative telomere signals. Telomere signals were determined by Flow-FISH and normalized to that of wildtype C57BL/6J mice (50 kb). **c,** TRF analysis. **d,** Body weight. **e,** Litter sizes. **f,** Testis weight. Each data point represents one animal. Means and SDs are shown.

To assess the stability of short telomeres in mice with the *hmTert* genes, homozygous *Tert*^*h/h*^ offspring from G4, G4.8, and G4.14 were incrossed for 13, 9, and 2 generations, respectively (Fig. 5a). Average telomere lengths in their progeny were measured by Flow-FISH. The results, depicted in Fig. 5b, revealed a gradual decrease in telomere length across successive generations. In G4 *Tert*^*h/h*^ mice, telomeres decreased from 60% to 30%, while in G4.8 *Tert*^*h/h*^ mice, the decline went from 34% to 24%. G4.14 *Tert*^*h/h*^ mice exhibited telomere lengths approximately 24% of the wildtype, and their offspring maintained telomeres at 22–23% of wildtype length during two successive generations of incrossing. These findings indicate that telomere length in *Tert*^*h/h*^ mice stabilized at a shortened but consistent ranges of 21–24% of wildtype mice, equivalent to an average telomere length of 10–12 kb, similar to reported leukocyte telomere lengths of 9.5 ± 0.7 kb and 10–11 kb in newborn humans ^2,22^. Additionally, TRF analysis confirmed the presence of discrete telomere bands between 15–20 kb and a human-like telomere smear (Fig. 5c). For G4.8(g-i) and G4.14(a-b) mice, the average lengths of telomere smears were 9–10 kb. Regardless of having shortened telomeres, these mice exhibited good health, as demonstrated by stable body weight, litter sizes, and testis weight (Figs. 5d–f).

**Extended Data Figure 4.**
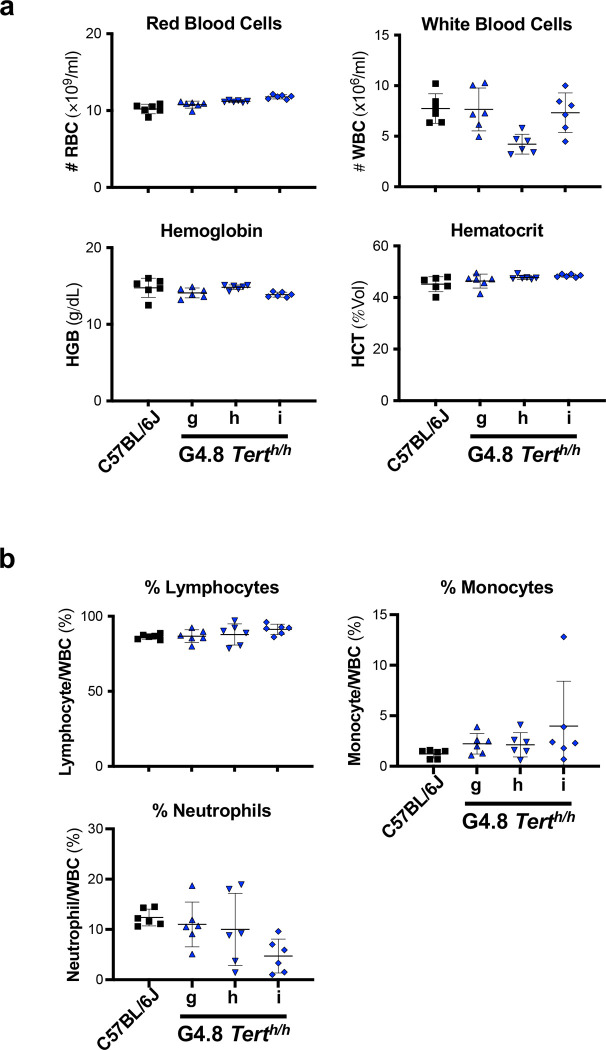
Blood cell counts of *Tert*^*h/h*^ mice. **a,** Whole blood counts. **b,** White blood cell counts in peripheral blood. Cells were stained using antibodies and analyzed by flow cytometry. Each data point represents one animal. Means and SDs are shown.

To further determine the health status of *Tert*^*h/h*^ mice with human-like telomeres, we performed hematology analysis on peripheral blood samples from G4.8g, h, and i mice aged 2–3 months. As shown in Extended Data Fig. 4, the result indicated normal red blood cell counts, hemoglobin, and hematocrit in all groups of mice. Although the average WBC count in G4.8h was lower than those of G4.8g, G4.8i, and wildtype mice, it still fell within the normal WBC range of physiological data for C57BL/6J mice published by the Jackson Laboratory ^23^. Within WBC populations, all groups of mice displayed normal percentages of lymphocytes, monocytes, and neutrophils, further supporting the notion that *Tert*^*h/h*^ mice with human-like telomeres maintain good health at early stages of their life.

### Comparing hmTert and mTert gene functions.

**Extended Data Figure 5.**
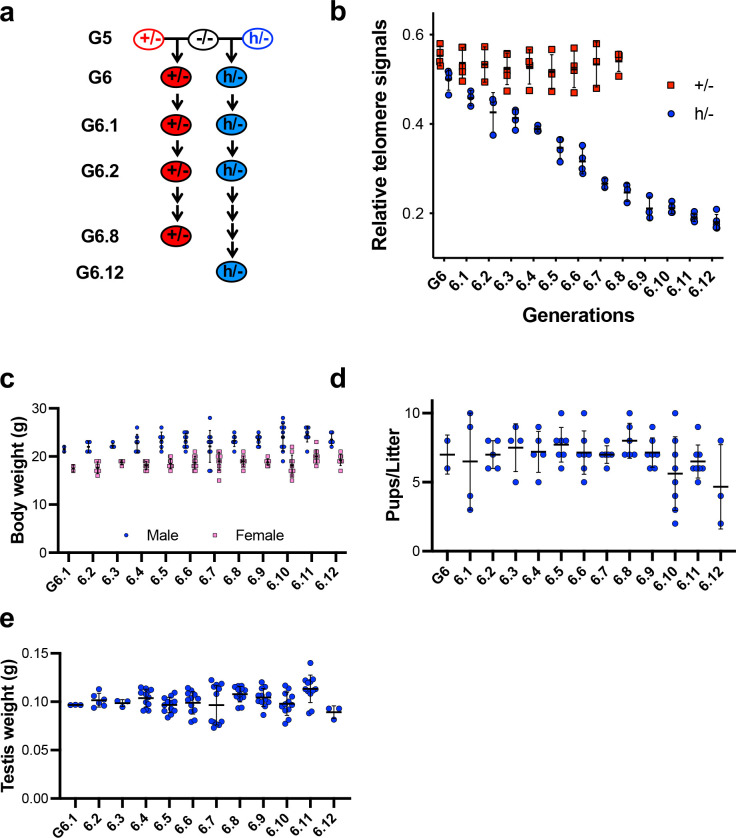
Comparison of *mTert* and *hmTert* alleles. **a,** Breeding strategy. G6 *Tert*^+/−^ and *Tert*^*h*/−^ mice from Fig. 3a were independently intercrossed. **b,** Relative telomere signals as determined by Flow-FISH and normalized to that of wildtype C57BL/6J mice. **c,** Body weight. **d,** Litter sizes. **e,** Testis weight. Each data point represents one animal. Means and SDs are shown.

In order to directly compare the functions of the *hmTert* and *mTert* genes and establish a new line of *Tert*^*h/h*^ mice, we conducted intercrosses using G6 *Tert*^*h*/−^ and *Tert*^+/−^ mice (Extended Data Fig. 5a). Consistent with our previous findings, telomere length in G6 *Tert*^*h*/−^ mice decreased from 55% to 18–19% of wildtype telomere length in C57BL/6J mice across 12 successive intercrosses (Extended Data Fig. 5b). In contrast, the average telomere lengths in G6 *Tert*^+/−^ mice remained stable at 55% of wildtype telomere length over eight intercrosses. This result aligned with a previous study in which *Tert*^+/−^ mice were intercrossed for 17 generations, stabilizing their average telomere length at approximately 50% of wildtype telomeres ^24^. Importantly, G6 *Tert*^*h*/−^ mice exhibited consistent litter sizes and development with appropriate body and testis weights across the entire breeding process (Extended Data Figs. 5c–e). Therefore, our data indicate that the *Tert* loci play a crucial role in regulating telomere length homeostasis, and the *hmTert* gene genetically determined short telomeres in mice.

### Decreased in vivo cell proliferation capacity in Tert^h/h^ mice.

**Figure 6.**
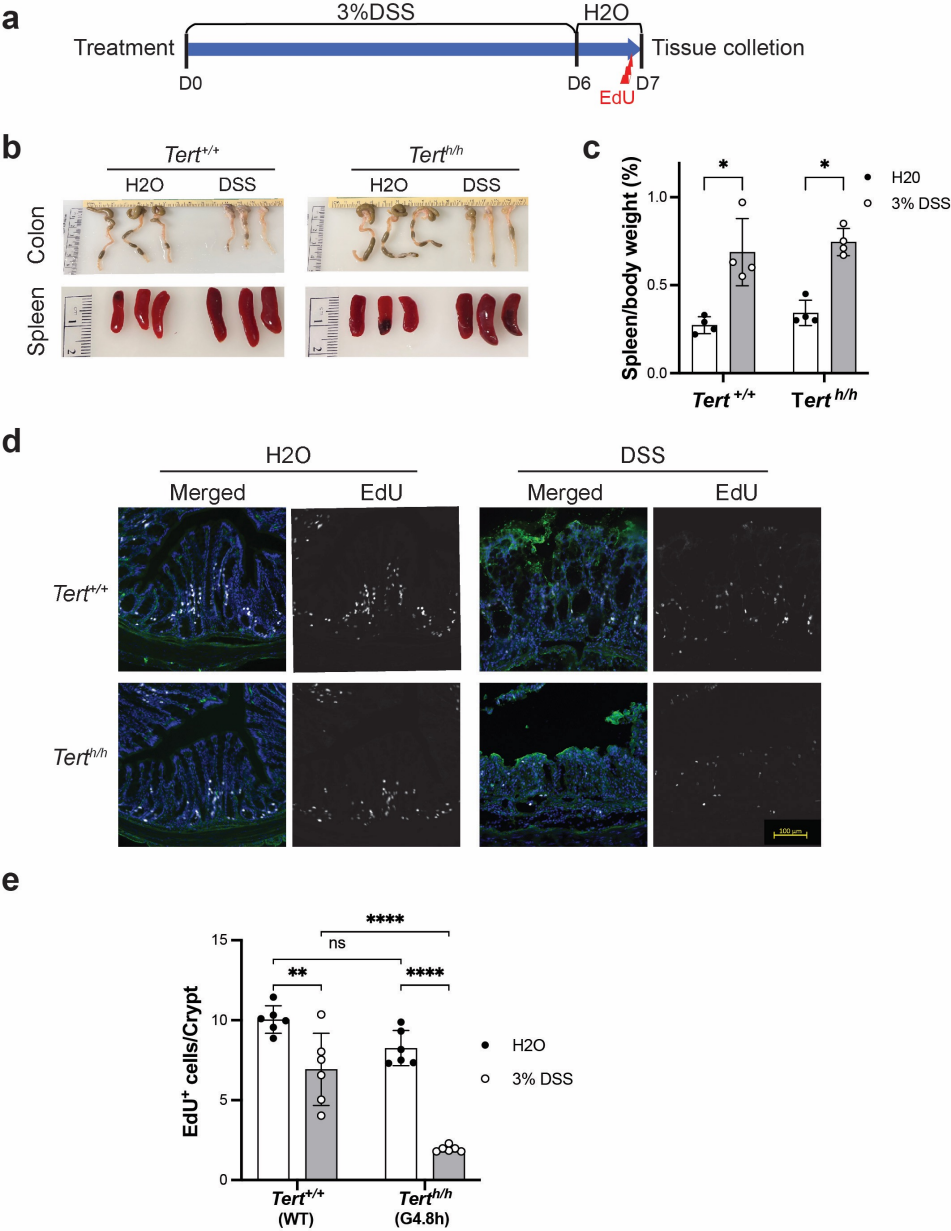
Dextran sulfate sodium (DSS)-induced colitis in mice. **a,** Experimental strategy. 7–8-month-old *Tert*^+/+^ (wildtype C57BL/6J) and *Tert*^*h/h*^ (G4.8h) mice were given drinking water with or without 3% DSS for 6 days, followed by 1 day of pure drinking water. Intraperitoneal EdU injection was performed 2 hours before tissue collection. **b,** Representative images of colons and spleens following DSS treatment. **c,** Spleen weight. Spleen weight was normalized to the body weight of each mouse. **d,** EdU staining of colon crypt sections. Colon tissues were labeled with anti-EdU (white) and E-cadherin (green) antibodies, as well as Hoechst dye for nuclear staining (blue). Images were captured using a Zeiss Image M2 microscope. Representative images are shown. **e,** Quantification of EdU-positive cells. Each data point represents the average number of EdU-positive cells per colon crypt in 30 crypts from one animal. ns, not significant; *, P < 0.05; **, P < 0.01; ****, P < 0.0001; N = 6; two-way Anova.

Ulcerative colitis is an inflammatory disease associated with telomere shortening and accelerated colon aging in human patients ^25^. G4.8h *Tert*^*h/h*^ mice remained in good health, and their gastrointestinal tracts appeared normal. As depicted in Fig. 6e, cellular proliferation, assessed by EdU incorporation, in the colons of G4.8h *Tert*^*h/h*^ mice showed a slight decrease, albeit statistically insignificant when compared to wildtype mice. To evaluate tissue renewal capacity under pathological conditions, mice were subjected to a 6-day dextran sodium sulfate (DSS) treatment to induce conditions akin to ulcerative colitis ^26,27^ (Fig. 6a). DSS treatment led to similar colon shortening and spleen enlargement in both *Tert*^+/+^ and *Tert*^*h/h*^ mice, indicating DSS-induced comparable inflammatory responses in both groups (Figs. 6b–c). The toxicity of DSS to colonic epithelial cells triggered a regenerative response upon toxin removal ^26^. In *Tert*^*h/h*^ mice, an average of about 2 EdU-positive cells per crypt cross-section were observed, significantly fewer than the average of 7 EdU-labeled cells per crypt cross-section in wildtype mice (Figs. 6d–e). These results suggested that, while *Tert*^*h/h*^ mice with human-like short telomeres maintained tissue homeostasis during development and adulthood under normal physiological conditions, tissue renewal was more limited under pathological conditions due to their short telomeres and absence of telomerase activity.

## Discussion:

While significant progress has been made in unraveling the mechanisms governing telomere maintenance and regulation, the genetic factors contributing to the variation in telomere length among species and individuals are still not fully understood. Moreover, the discrepancies in telomerase expression and telomere length between mice and humans pose significant challenges for studying human cancer and age-related diseases using mouse models. To overcome this obstacle, we engineered a humanized version of the *mTert* gene, known as *hmTert*, by replacing the 5’IR and introns 2 and 6 with their human counterparts in mouse ESCs ^11,28^. In this report, we generated and characterized mouse lines with germline *hmTert* alleles. Our findings demonstrate that the regulation of the *hmTert* gene closely mimics that of the *hTERT* gene during development and in adult tissues and the *hmTert* gene reset mouse telomere to human length. Given the inability of hTERT protein to function with mTerc and other telomerase accessory proteins ^29,30^, the generation of a similar mouse model using alternative approaches, such as complementing a *mTert* knockout allele with a transgenic *hTERT* gene, is not feasible ^7^.

Telomere homeostasis is regulated by genes encoding telomerase, the shelterin protein complex, and other telomere-protecting proteins, such as Rtel helicase ^31,32^. The shelterin proteins exhibit structural and functional conservation between mice and humans, with the exception of POT1. Mice possess two paralogs, mPot1a and mPot1b, and they both bind TPP1 and perform the same overall functions as the single human POT1 protein ^33^. Our data indicate that humanization of the *mTert* gene resets equilibrium telomere length. During both intercrosses of *Tert*^*h*/−^ mice and incrosses of *Tert*^*h/h*^ mice, average telomere lengths progressively shortened until reaching 9–10 kb and 10–12 kb, respectively. The new equilibrium point of telomere length for *Tert*^*h/h*^ mice is similar to that observed in human telomeres, resulting a mouse strain with humanized telomerase expression and telomere length. These *Tert*^*h/h*^ mice, named HuT mice, possess a C57BL/6J genetic background.

Our data unequivocally establish the *Tert* gene as a primary genetic determinant of telomere length in mice. A fundamental difference between the *hTERT* and *mTert* genes lies in the rich context of repetitive sequences that emcompassing the hTERT promoter, including transposable elements (TEs) and variable number tandem repeats (VNTRs), primarily within the 5’ IR and intron 2 (Fig. 1a). These elements play a crucial role in maintaining stringent hTERT repression in most adult tissues while allowing its expression in key organs, such as the thymus and gonads ^11, 34–36^. Although the precise molecular basis for the shorter telomeres in *Tert*^*h/h*^ and *Tert*^*h*/−^ mice compared to *Tert*^+/+^ or *Tert*^+/−^ mice warrants further investigation, we have observed the *hmTert* gene more effectively rescues telomere dysfunction-induced seminiferous tubule atrophy compared to the *mTert* allele. This is particularly noteworthy, even with the lower *hmTert* mRNA expression in the testis compared to mTert mRNA (Fig. 1c). It is conceivable that the higher expression of *mTert* throughout germ cell development results in longer telomeres in progeny. Additionally, our previous data have indicated that the hTERT promoter activity is especially high in elongating spermatids ^8^. These cells are undergoing dramatic epigenetic reprogramming, involving the replacement of canonical histones with protamines and extensive DNA methylation ^37^, and exhibit elevated telomerase activity ^38^. The robust expression of *hmTert*/*hTERT* in these cells likely contributes to maintaining telomere integrity and germline cell survival during this crucial stage, allowing haploid germ cells with short telomeres to develop into sperm. This also provides a plausible explanation for the efficient rescue of telomere dysfunction by *hmTert* in the testis. Further investigation into hmTert/hTERT expression in the testis cells may yield deeper insights into this phenomenon at the cellular level.

Mice with genotypically wildtype characteristics but short telomeres have been reported previously ^21,24,39^. These mice were obtained through crossing with wild-derived Cast/EiJ mice and/or homozygosity induction following extensive intercrosses of heterozygous *Terc*^+/−^ mice. Another approach involved a mutation in the *Rtel* gene ^40^. Although telomeres in these mice were shorter than those in wildtype C57BL/6 mice, they were still substantially longer than human telomeres. Furthermore, telomerase expression remained widespread in somatic tissues of these mice. In contrast, HuT mice not only have human-like short telomeres but also possess little to no telomerase activity in most adult tissues, making them a novel model for studying human diseases, particularly cancer and age-related degenerative diseases. Additionally, the rate of telomere shortening per generation was higher during *Tert*^*h*/−^ intercrosses than in *Tert*^*h/h*^ incrosses. *Tert*^*h*/−^ offspring also had shorter telomeres than their *Tert*^*h/h*^ siblings during intercrosses. These data indicate that the *hmTert* gene is haploinsufficient, rendering *Tert*^*h*/−^ mice an ideal model for dyskeratosis congenita, a human genetic syndrome resulting from genetic mutations in telomerase genes.

The average telomere lengths in HuT mice closely resemble those observed in humans. TRF analyses revealed that telomere signals in *Tert*^*h*/−^ and *Tert*^*h/h*^ mice consist of discrete bands measuring 15–20 kb, accompanied by a lower-molecular-weight smear reminiscent of human telomeres. Importantly, these discrete telomere bands are distinct from interstitial telomere bands, as their sizes and intensities vary among individual mice. Instead, these bands likely represent ultra-long telomeres present in C57BL/6J mice that have yet reached the length of human telomeres. Given that the shortest telomeres are prone to dysfunction and can limit cell proliferation, the presence of a few longer telomeres may have minimal impact on aging and tumorigenesis. Moreover, it is expected that these long telomeres will continue to shorten over successive generations during the breeding of these mice.

It is widely acknowledged that the shortest telomeres, rather than the average telomere length, play a pivotal role in maintaining chromosomal stability and triggering replicative senescence ^41^. Therefore, it comes as no surprise that *Tert*^*h/h*^ mice with very short telomeres (HuT mice) remain in good health during the early stages of life. Previous research has reported variations in the rates of telomere shortening among different mammalian species ^3^. While human peripheral blood cell telomeres shortened at a chronological rate of 31–72 bp/year, C57BL/6 mice experienced a much more rapid shortening, at a rate of 7000 bp/year ^42^. Consequently, telomere reserve in HuT mice may impose limitations on cell proliferation and tissue renewal. Indeed, our experiments revealed that colonocyte proliferation was constrained in HuT mice under a DSS-induced ultracerative colitis condition. Telomere attrition has been implicated as a counting mechanism of cellular replicative senescence in vitro and organismal aging in vivo. Our objective is to delve into the aging process of HuT mice and explore whether their cells undergo replicative senescence.

The development of HuT mice has the potential to catalyze a paradigm shift in cancer and aging research using mouse models. Firstly, the presence of short telomeres in HuT mice is likely to impede tumor cell proliferation during cancer development, as dysfunctional telomeres can induce chromosomal instability and significantly impact the course of cancer progression. Secondly, the limited telomere reserve in HuT mice and humans can also exert a profound influence on the tumor microenvironment and the host immune system. Recent research has indicated that host T cell immune deficiency, rather than tumor cell chromosome instability, predisposed patients with short telomere syndromes to squamous cancers ^43^. Thus, telomere-driven immune senescence may contribute to the high tumor incidences among aged humans and HuT mice. Thirdly, chemotherapy drugs can induce DNA damage in both normal cells and tumor cells. Considering that tissues and cells with short telomeres are inherently more susceptible to such damage, this susceptibility could synergize with the cytotoxic effects of cancer drugs, particularly in older humans and HuT mice. Consequently, HuT mice may be a better preclinical model for evaluating drug toxicity. Taken together, HuT mice are expected to serve as an enhanced mouse model for investigating tumorigenesis, thereby providing invaluable insights into cancer development and potential therapies.

In conclusion, the process of telomere attrition plays a pivotal role in the development of various human diseases. To gain a deeper understanding of the fundamental disparities in telomerase regulation and telomere homeostasis between mice and humans, we have developed genetically engineered HuT mice. Our data demonstrated that the *hmTert* allele is a functional gene and contributes to establishing a telomere length setpoint in mice that closely resembles that of humans. These mice exhibit humanized telomerase expression and telomere homeostasis, and ongoing investigations are focused on studying their aging process and susceptibility to age-related diseases. The development of HuT mice represents an opportunity to address previously unattainable inquiries concerning human aging and cancer, opening new avenues for scientific exploration.

## Methods:

### Generation of mice with hmTert alleles.

The engineering of the *hmTert* gene (Fig. 1a) in mouse ESCs (*Tert*^*h*/+^) was previously reported ^1^. The ESCs (G4, 129xC57BL/6)^2^ were injected into blastocysts of C57BL/6J albino hosts. The resulting male chimera mice were bred with C57BL/6J female mice. Mice with a germline *hmTert* allele (F0) were obtained and crossed again with C57BL/6J mice to generate F1 *Tert*^*+/h*^ mice with 88% C57BL/6J background. The F1 *Tert*^*+/h*^ mice were backcrossed with C57BL/6J mice for three generations before *Tert*^*h*/+^ mice were used for mRNA expression analyses. *Tert*^*+/h*^ mice were also crossed with *Tert*-knockout (*Tert-KO*) mice ^3^. All animal experiments were approved by the Institutional Animal Care and Use Committee in accordance with the NIH Guide for Care and Use of Animals.

### Gene expression and telomerase activity.

mRNA expression analyses were performed as previously described ^4^ and data were normalized to 18S ribosomal RNA. Primer sequences are provided in Extended Data Table 1. Telomerase activities were determined using a modified telomeric repeat amplification protocol (TRAP) assay ^1^. Tissues and cell extracts were adjusted to same concentration and 0.5μg samples were used in each reaction. ESCs (*Tert*^*h/h*^, *Tert*^+/+^, and *Tert*^*+/h*^) served as positive controls.

### Telomere length measurements.

Telomere lengths were measured using two independent methods. TRF analysis was described previously ^1^. Genomic DNAs were digested with Hinf I and Rsa I, and subjected to pulsed-field gel electrophoresis using CHEF-DR III Pulsed field Electrophoresis Systems (for telomeres over 20-kb) or regular 0.6% Agarose gel (for telomeres less than 20-kb), followed by Southern blotting using a (TTAGGG)_3_-Biotin probe. Probe signal was developed with Chemiluminescent Nucleic Acid Detection Module Kit (Thermo Fisher). Telomere lengths were also measured by Flow-FISH. Telomere signals were detected by hybridization to FAM-(CCCTAA)_3_ oligonucleotide and converted to arbitrary units of molecular equivalents of soluble fluorescence.

### T cell culture and stimulation.

CD4^+^ and CD8^+^ T cells were isolated from splenocytes using MojoSort^™^ T cells isolation kits (Biolegend, USA). Briefly, spleens were collected in RPMI-1640 medium (Gibco, USA) as soon as mice were sacrificed. The splenocytes were isolated through spleen crushing and filtration using a 70 μm strainer. Red blood cells were lysed with 1x RBC lysis buffer, splenocytes were incubated with biotin-antibody cocktails on ice for 20 mins, and streptavidin nanobeads beads were added for 5 mins, followed by pulldown of non-T cells. 6-well plates were coated by 10μg/ml anti-mouse CD3e antibody in PBS for 2 hours at 37°C. The isolated CD4 or CD8 T cells were stimulated in CD3e antibody coated plates, together with added 2 μg/ml anti-mouse CD28 antibody for 24 h. 10 ng/ml IL-2 was added at 48, 72 and 96 h and activated T cells were examined for cell proliferation using Click-iT^®^ Plus EdU Flow Cytometry Assay Kit (ThermoFisher, USA) or harvested for gene expression analyses.

### Histological analyses.

Fresh intestines were fixed in 10% formalin solution for 48 h, embedded in paraffin, sectioned at 5 μm, and stained with hematoxylin and eosin (H&E). Mouse testes were fixed in Bouin’s fixative for 3 days and similarly processed. Slides were evaluated for aberrant seminiferous tubules in a blinded fashion and at least three mice of each experimental group were analyzed.

### Hematological analysis.

The whole blood samples were collected from submandibular veins of 3–6 months mice. Numbers of WBCs, RBCs, platelets, lymphocytes, neutrophils, and monocytes were determined on a Hemogram Analyzer (Abaxis HM5).

### FACS analyses.

Peripheral blood, splenocytes, and femur bone marrow (BM) were collected from mice of 3–6 months. Peripheral blood was washed twice with PBS by centrifugation in the presence of heparin, followed by removing RBCs with 1x RBC lysis buffer (Thermo Fisher, USA). WBCs were counted using hemocytometers, stained with CD4, CD8, and CD19 antibodies, and analyzed by flow cytometry. Splenocytes and bone marrow cells, flushed from femurs by syringes with 20 ml PBS buffer, were similarly processed for cell counting, FACS staining, and analyses.

### Dextran sulfate socium treatment.

Female mice of 7–8 months of age were subjected to a 6-day period of 3% Dextran sulfate sodium (DSS) (Cat. 160110, MP biomedical) administration in drinking water, followed by a day of regular drinking water before tissue collection. Body weight of treated mice were monitored. For EdU labeling, 150μl of 10 mg/ml EdU solution was injected intraperitoneally into mice two hours before sacrifice. Paraformaldehyde-fixed tissues were embedded in OCT matrix. EdU was detected using the Click-iT Plus EdU Cell Proliferation Kit (Alexa Fluor^™^ 594, C10639, ThermoFisher). Tissue sections were stained overnight with an anti-E-Cadherin antibody (1:250, Cat. 610181, BD Biosciences), followed by incubation with FITC-conjugated secondary antibody and Hoechst 33342 (1:2000). Images were captured using a Zeiss AXIO Image M2 microscope and processed using FIJI and Adobe Photoshop 2023.

### Statistical analysis.

GraphPad Prism 10 was used for statistical analyses, including two-tailed student’s t tests for comparisons of gene expression, one or two-way Anova for comparisons of body and organ weights and cell numbers among different experimental groups, and logrank tests for mouse survival curves.

## Extended Data

**Extended Data Table 1.** Oligonucleotide Sequences (5’–>3’)

TRAP assay			Amplicon
TS	AATCCGTCGAGCAGAGTT		
NT	ATCGCTTCTCGGCCTTTT		
TSNT	AATCCGTCGAGCAGAGTTAAAAGGCCGAGAAGCGAT		
ACX	GCGCGGCTTACCCTTACCCTTACCCTAACC		
			
**Oligos for mouse genotyping**			
hmTert	ATCTCCTGCAGGTTTGCTGT	AGAAACGCATCACAGACACG	363
mTert	GGTCACCAGGCCCTCGGTGATCTGGATGTGGCATGTC CTTTACAAGGATCACCGGTTCGT	TGGAATGTGCTCTCTGTGATG	437
mTert-KO	CATTGTGGATCTGAGGTGAGTC	CGCCTTCTTGACGAGTTCTT	467
			
**Quantitative RT-PCR primers**			
hmTert	TGTGGAAGATGAAGGTCGAA	CCACGTATGTGTCCATCAGC	135
mTert	TGAAAGTAGAGGATTGCCACTG	CCACGTATGTGTCCATCAGC	126
hTERT	GAACATGCGTCGCAAACTCTTTGG	TGCAGCAGGAGGATCTTGTAGATG	
hmTert&mTert	GCAGGAACTGATGTGGAAGA	CCACGTATGTGTCCATCAGC	For intestine qRT-PCR in h/−,+/−,−/−,+/+ mice
p16	GCTGGGTGGTCTTTGTGTA	TTAGCTCTGCTCTTGGGATTG	
p21	CTGAGCGGCCTGAAGATT	ATCTGCGCTTGGAGTGATAG	
Ki67	CTGTCACTCCAGATCAGAACTC	GGAGGCAGTCTTCATAGTCTTC	
PCNA	CGAAGCACCAAATCAAGAGAAAG	CACCCGACGGCATCTTTATTA	
TNF-α	CCCCAAAGGGATGAGAAGTT	CACTTGGTGGTTTGCTACGA	
IL-6	GTTCTCTGGGAAATCGTGGA	TTCTGCAAGTGCATCATCGT	
18s RNA	TAGAGGGACAAGTGGCGTTC	CGCTGAGCCAGTCAGTGT	104

## Data Availability

Raw data related to any experiments presented in this article are available from the corresponding author upon request.
